# Association between red blood cell count and cortical functional connectivity in type 2 diabetes mellitus patients residing at high altitude: resting-state fMRI study in the Tibetan Plateau

**DOI:** 10.3389/fendo.2026.1931880

**Published:** 2026-10-14

**Authors:** Jinli Meng, Yingxue Gao, Hailong Li, Li Feng, Xiangwei Li, Zan Zhou, Xin Hu, Hengyan Li, Yuanyuan He, Yongyue Guo, Xiaoyan Luo, Wanlin He, Yunhong Wu, Xiaoqi Huang

**Affiliations:** 1 Department of Radiology, Huaxi MR Research Center (HMRRC), Institute of Radiology and Medical Imaging. West China Hospital of Sichuan University, Chengdu, Sichuan, China; 2 Psychoradiology Key Laboratory of Sichuan Province, West China Hospital of Sichuan University, Chengdu, Sichuan, China; 3 Department of Radiology, Hospital of Chengdu Office of The People’s Government of Xizang Autonomous Region, Chengdu, Sichuan, China; 4 Department of Endocrinology and Metabolism, Hospital of Chengdu Office of The People’s Government of Xizang Autonomous Region, Chengdu, Sichuan, China; 5 Xiamen Key Lab of Psychoradiology and Neuromodulation, Department of Radiology, West China Xiamen Hospital of Sichuan University, Xiamen, Fujian, China

**Keywords:** amygdala, functional connectivity, high altitude, hippocampus, red blood cell, type 2 diabetes mellitus

## Abstract

**Background:**

Type 2 diabetes mellitus (T2DM) is associated with cognitive dysfunction and emotional disturbances, in which the hippocampus and amygdala are key limbic structures. We investigated hippocampal and amygdala functional connectivity (FC) in Tibetan patients with T2DM residing in a high-altitude hypoxic environment and examined associations with clinical and biochemical measures.

**Methods:**

We included 87 Tibetan patients with T2DM and 63 healthy controls (HC) from the Tibetan Plateau. Seed-based resting-state FC analysis was used to compare whole-brain hippocampal and amygdala FC between groups. Associations between altered FC and biochemical indices, emotional symptoms, and cognitive performance were assessed using Pearson or Spearman correlation analyses.

**Results:**

Compared with HC, Tibetan patients with T2DM showed increased FC between the right hippocampus and the left dorsolateral prefrontal cortex (DLPFC)/superior frontal gyrus and between the left amygdala and the right orbitofrontal cortex (OFC)/right DLPFC. Within the T2DM group, hippocampal FC was positively associated with red blood cell (RBC) count and visuospatial memory scores, whereas amygdala FC was positively associated with visuospatial memory scores and Mini-Mental State Examination scores.

**Conclusion:**

Tibetan patients with T2DM showed increased hippocampal and amygdala FC, which was associated with RBC count and memory-related cognitive performance. No significant associations were observed between altered FC and blood glucose measures. These findings characterize limbic-prefrontal connectivity alterations and their clinical associations in this high-altitude Tibetan T2DM cohort.

## 1 Introduction

Type 2 diabetes mellitus (T2DM) accounts for 90-95% of diabetes cases, affects ~450 million adults worldwide, and increases cognitive impairment and dementia risk (1). In 2013, China had the largest diabetes population and second-highest diabetes-related expenditure globally (2). Among Chinese populations, Tibetans have higher T2DM incidence than Han Chinese (3), a disparity potentially explained by socioeconomic and behavioral factors. Tibetans reside at high altitudes characterized by hypoxia, which impairs cortical functions (attention, perception, memory, motor initiation) and increases depression risk (4, 5). Fasting plasma glucose (FPG) levels increase with elevation (6), but the neural pathophysiology of T2DM remains poorly understood, particularly in Tibetans.

T2DM is a risk factor for progression from mild cognitive impairment (MCI) to dementia (7). The hippocampus and amygdala are key limbic structures linked to cognitive impairment and emotion dysregulation in T2DM. Both exhibit disrupted neuropeptide and hormone secretion in diabetic rodent models (8, 9). Structural alterations in these regions have been demonstrated in T2DM patients and Tibetan populations (10–12), and disrupted functional connectivity between them has been confirmed by resting-state functional connectivity (rsFC) studies (13). Moreover, altered hippocampal (14) and amygdala (15) connectivity partially mediate the relationship between cognitive dysfunction and depressive mood in T2DM patients (16).

Resting-state functional connectivity is derived from blood oxygen level-dependent (BOLD) signals, which are modulated by blood supply and metabolic status (14, 15). Chronic high-altitude environments (typically >2500 m in altitude) are characterized by hypobaric hypoxia, accompanied by reduced partial pressure of inspired oxygen, enhanced ultraviolet radiation, and large diurnal temperature variation (17). Owing to hypoxic exposure at high altitude, native Tibetan populations exhibit adaptive elevations in hemoglobin, red blood cell (RBC) counts, and metabolic parameters, a process partly regulated by high-altitude adaptive genetic variants such as EPAS1 (endothelial PAS domain protein 1) and EGLN1 (4). RBC count is an independent risk factor for elevated glycated hemoglobin (HbA1c) (18), whereas hyperglycemia and hyperlipidemia are well-recognized risk factors for cognitive impairment in patients with T2DM (19). The hypoxic environment unique to high altitude can substantially alter RBC levels and cerebral hemodynamics, suggesting that the potential mechanisms underlying brain functional alterations in T2DM patients at high altitudes may differ from those in lowland patients. Previous neuroimaging studies of high-altitude native or long-term resident populations have confirmed associations between peripheral physiological biomarkers and resting-state functional connectivity; in migrants to high altitude and native Tibetans, hemoglobin and hematocrit levels are associated with interhemispheric functional connectivity and the topological properties of large-scale brain networks (20). However, most prior high-altitude neuroimaging studies enrolled healthy individuals, and few have examined the associations between hematological biomarkers and limbic-cortical functional connectivity in patients with chronic metabolic diseases such as T2DM, representing an important knowledge gap that the present study aims to fill. Therefore, Tibetans provide a natural model for investigating the relationship between RBC counts and hippocampal/amygdala FC in diabetes.

In this study, we investigated intrinsic rsFC of the limbic network in Tibetan T2DM patients and explored associations between altered connectivity and clinical profiles, including anthropometrics, blood pressure, body mass index (BMI), plasma glucose, lipids, complete blood count, biochemical indices, and neuropsychological scores (memory, attention, executive function). We hypothesized that hippocampal and amygdala FC would be altered in patients with T2DM and that these alterations would be associated with RBC count.

## 2 Methods

### 2.1 Data source and study population

This cross-sectional observational study enrolled Tibetan plateau patients with T2DM from the Department of Endocrinology and plateau healthy controls (HC) from the Health Examination Center at Hospital of Chengdu Office of The People’s Government of Xizang Autonomous between January 2020 and December 2025. All participants were Tibetan residents who habitually resided in high-altitude areas at elevations ≥ 3500 m.Written informed consent was obtained from all participants. T2DM was defined according to the 2014 American Diabetes Association criteria. Participants were classified as having T2DM if they met the diagnostic glycemic criteria at enrollment (FPG ≥7.0 mmol/L or 2-h OGTT glucose ≥11.1 mmol/L) or had a previously established clinical diagnosis of T2DM. Participants with a prior diagnosis of T2DM were classified as having T2DM regardless of their glucose levels at enrollment, including those receiving glucose-lowering treatment. Patients with hypoglycemia (blood glucose < 3.9 mmol/L) or severe hyperglycemia (blood glucose > 33.3 mmol/L) during hospitalization were excluded.

Control participants had no known history of diabetes and were not receiving glucose-lowering medications. Glycemic screening information was available for all controls: 36 underwent glycemic testing as part of the present study, whereas the remaining controls reported having undergone glycemic screening within the preceding 6 months with no abnormal results and therefore did not undergo repeat standardized glycemic testing at enrollment, particularly HbA1c and/or OGTT.

Control participants were recruited using group-level frequency matching for age and sex. Specifically, controls were selected to approximate the distribution of the T2DM group across prespecified age categories (30–40, 40–50, 50 -60,and 60–70 years) and sex. Individual participants were not pair-matched.

The exclusion criteria were: (1) a history of cancer, coronary heart disease, stroke, chronic obstructive pulmonary disease, or heart failureb; and (2) contraindications to MRI scanning.

### 2.2 Clinical assessment

#### 2.2.1 Physiological and biochemical indices

Physiological and biochemical data were collected when available, including weight, height, blood pressure, BMI, plasma glucose (HbA1c and FPG), blood lipids (total cholesterol [TC], high-density lipoprotein [HDL], low-density lipoprotein [LDL], very low-density lipoprotein [VLDL], and triglyceride [TG]), and complete blood count (CBC) (red blood cell count, hemoglobin [HGB], hematocrit [HCT], and mean corpuscular volume [MCV]). Some laboratory measures were unavailable, particularly among controls, because not all biochemical tests were performed as part of the study assessments.All measurements were obtained, via standard laboratory testing after an overnight fast of at least 8 hours. the reference ranges for the parameters are based on the values calibrated by that instrument. A normal RBC count is defined as follows: 4.3-5.8 × 10¹²/L for males and 3.8-5.1 × 10¹²/L for females (21).

#### 2.2.2 Emotional symptoms index

All participants completed questionnaires assessing emotional symptoms, including the Pittsburgh Sleep Quality Index (PSQI), Patient Health Questionnaire-9 (PHQ-9), Generalized Anxiety Disorder Scale (GAD-7), Self-rating Depression Scale (SDS), and Self-rating Anxiety Scale (SAS) (see eMethods 1 in **Supplement 1**).

#### 2.2.3 Cognitive function

All participants underwent neuropsychological tests evaluating cognitive performance, including general cognitive function (Montreal Cognitive Assessment [MoCA, Beijing edition] and Mini-Mental State Examination [MMSE]), processing speed (Digit Span Test [DST] forward and backward), and memory function (logical memory test and visuospatial memory test) (see eMethods 2 in **Supplement 1**).

### 2.3 Magnetic resonance imaging data acquisition

All MR images were acquired within 1 week after participants left the plateau at West China Hospital, Sichuan University, Chengdu, China (elevation approximately 500 m), using a 3.0-T Philips Achieva MRI scanner with an 8-channel phased-array head coil. The exact number of days between descent and MRI acquisition was not systematically recorded. During scanning, participants were instructed to remain quiet, keep their eyes closed, and stay awake. Conventional T2-weighted and fluid-attenuated inversion recovery images were acquired to exclude visible brain lesions. Scan parameters were as follows: resting-state functional BOLD images: TR/TE = 2000/30 ms, FA = 90°, thickness = 3.7 mm, slices = 38, no gap, FOV = 220 × 220 mm^2^, matrix = 64 × 64, voxel size = 3.43 × 3.43 × 3.43 mm^3^, and 240 volumes.

### 2.4 Imaging preprocessing

Functional data were analyzed using DPABSF (http://www.rfmri.org/dpabi, version 4.4) and Statistical Parametric Mapping (SPM8, http://www.fil.ion.ucl.ac.uk/spm) running on MATLAB. The preprocessing steps were as follows: (1) First 10 time point removed: Removing the first 10 time points ensures signal stability. (2) Slice Timing and Head motion correction: Since all participant’s functional images were acquired at different time points within a single repetition, it is necessary to correct for temporal differences between image slices. (3) Image registration, segmentation, and standardization: The corrected functional images were registered to the corresponding T1 structural images using the Differential Nonlinear Image Registration Algorithm (DARTEL), they were segmented into gray matter, white matter, and cerebrospinal fluid, and then the segmented images were standardized to the Montreal Neurological Institute (MNI) standard space, and the functional image voxels are resampled to 3×3×3 mm³. (4) Smoothing: To control noise and effects caused by residuals resulting from the averaging of functional and structural data across individuals, the images are smoothed using a Gaussian smoothing kernel with a full width at half maximum (FWHM) of 6 mm. (5) Linear Detrending: To remove the effects of instrument instability and reduce signal drift. (6) Filtering: To remove the effects of physiological noise such as respiration and heartbeat; commonly used low-frequency filters target the range of 0.01-0.08 Hz. To minimize head motion effects, we performed scrubbing with a framewise displacement (FD) threshold > 0.3 mm. MRI data were excluded if: (1) spatial movement exceeded 2 mm or 2° in any direction, (2) mean FD was > 0.3 mm, or (3) scrubbed data represented < 70% of time points. Based on these criteria, 5 T2DM patients and 1 HC were excluded from further analysis.

### 2.5 Seed-based FC analysis

Whole-brain FC maps for the bilateral hippocampus and amygdala were generated by defining seed regions of interest (ROIs) based on the Automated Anatomical Labeling (AAL) atlas and calculating voxel-wise correlations between each seed and all other brain voxels. The resulting correlation coefficients were then converted to z scores using Fisher’s r-to-z transformation.

## 3 Statistical analysis

### 3.1 Group comparison

All statistical analyses were performed using SPSS 24.0. We compared demographic and clinical characteristics between patients with T2DM and healthy controls (HC). Continuous variables were compared via two-tailed t-tests, while sex distribution was compared using the chi-square test. Statistical significance was defined as p < 0.05.

Analysis of covariance (ANCOVA) was performed using SPM8 to identify brain regions showing significant group differences in bilateral hippocampal and amygdalar FC. Age, sex, and head motion (mean FD) were included as covariates. The significance threshold was set at p < 0.001 (uncorrected) at the voxel level, and cluster size > 100 at the cluster level. Finally, the threshold was set to p < 0.05, with family-wise error (FWE) correction at the cluster level.

### 3.2 Correlation analysis

To investigate relationship between altered hippocampal and amygdala FC and demographic and clinical variables, we used Pearson correlation analyses for continuous variables and Spearman rank correlations for dichotomous variables. To account for multiple testing, the Benjamini–Hochberg false discovery rate (FDR) procedure was applied within clinically defined correlation families. Both uncorrected P values and FDR-adjusted q values were calculated. Given the exploratory nature of these analyses, associations with an uncorrected *P* < 0.05 are reported as nominally significant, whereas associations with an FDR-adjusted *q* < 0.05 are considered statistically significant after correction for multiple comparisons. Complete results, including the sample size for each analysis, are provided in **eTable S3** in **Supplementary 1**.

## 4 Results

### 4.1 Demographics

After imaging quality control and age- and sex-based matching, we ultimately enrolled 87 patients with T2DM (FPG, 7.76 ± 2.26 mmol/L; HbA1c, 9.64 ± 2.20%) and 63 HC.

A higher proportion of T2DM patients had a history of smoking (p < 0.05). No other significant demographic differences were observed between the two groups (**Table 1**).

**Table 1:** Demographic, clinical data, psychological symptoms and neuropsychiatric test results in T2DM patients and HC.

Characteristics	T2DM group (n, mean ± SD)	HC group (n, mean ± SD)	p value
	n = 87	n = 63	
Age (years) ^b^	53.59 ± 8.99 (33-70)	50.64 ± 10.11 (23-71)	0.067
Sex (M/F) ^a^	50/37	27/36	0.077
Altitude (meters) ^b^	86, 3595.35 ± 300.06	61, 3637.38 ± 438.63	0.518
Illness duration (years)	9.24 ± 7.30	NA	NA
Smoking history (yes/no) ^a^	44/43	14/49	<0.001*
Drinking history (yes/no) ^a^	34/53	29/34	0.395
HbA1c (%)	83, 9.64 ± 2.20	NA	NA
FPG (mmol/L)	83, 7.76 ± 2.26	NA	NA
BMI (kg/m^2^) ^b^	87, 25.37 ± 3.80	63, 24.77 ± 4.74	0.401
Blood pressure (mmHg)			
Systolic blood pressure (mmHg) ^b^	84,127.99 ± 17.56	59, 121.91 ± 12.89	0.027*
Diastolic blood pressure (mmHg) ^b^	84, 81.46 ± 9.69	59, 76.64 ± 8.20	0.005*
Cholesterol			
TC (mmol/L) ^b^	83,4.34 ± 0.92	28, 4.34 ± 1.05	0.976
HDL (mmol/L) ^b^	83,1.11 ± 0.21	28,1.27 ± 0.39	0.049*
LDL (mmol/L) ^b^	83,2.61 ± 0.76	33,2.61 ± 0.80	0.962
VLDL (mmol/L) ^b^	83,0.62 ± 0.36	33,0.47 ± 0.20	0.005*
TG (mmol/L) ^b^	83,1.61 ± 0.95	27,1.06 ± 0.47	0.005*
CBC			
RBC (10^12^/L) ^b^	82,5.09 ± 1.07	36,4.69 ± 0.54	0.037*
HGB (g/L) ^b^	82,152.76 ± 26.80	36,142.97 ± 21.80	0.056
HCT (%) ^b^	82,46.20 ± 8.55	36,43.31 ± 5.81	0.067
MCV (fL) ^b^	82,91.34 ± 6.46	36,92.71 ± 8.07	0.329
MCH (pg) ^b^	82,30.26 ± 2.86	36,30.48 ± 3.18	0.723
MCHC (g/L) ^b^	82,331.0 ± 13.86	36,329.19 ± 11.69	0.497
Living quality			
PSQI ^b^	73,5.96 ± 3.88	57,6.47 ± 4.05	0.463
PHQ-9 ^b^	69,2.97 ± 3.75	58,3.33 ± 4.18	0.613
GAD-7 ^b^	71,2.43 ± 3.49	58,2.36 ± 3.69	0.909
Anxiety and depression			
SDS ^b^	71,32.46 ± 7.17	58,33.38 ± 8.62	0.512
SAS ^b^	69,33.72 ± 7.69	58,32.71 ± 9.16	0.497
Cognitive performance			
MMSE ^b^	63,24.22 ± 4.50	56,25.04 ± 4.35	0.319
MoCA ^b^	60,20.75 ± 5.38	55,21.22 ± 4.94	0.629
Processing speed			
DST-forward ^b^	73,7.36 ± 1.68	6.95 ± 2.13	0.222
DST-backward ^b^	73,3.84 ± 1.24	3.73 ± 1.45	0.639
Logical memory			
Logical memory test-immediate ^b^	73,8.90 ± 4.39	8.82 ± 4.69	0.943
Logical memory test-delayed ^b^	73,7.14 ± 4.40	7.36 ± 4.54	0.845
Visuospatial memory			
Visuospatial memory test-immediate ^b^	69,3.35 ± 2.53	6.35 ± 2.98	<0.001*
Visuospatial memory test-delayed ^b^	69,3.05 ± 2.53	6.44 ± 3.01	<0.001*

The data are presented as the mean ± SD and n. NA, not applicable. ^a^The statistical analyses were performed by the χ2 test. ^b^The statistical analyses were performed by two independent sample t tests. *p value < 0·05. HbA1c, glycosylated hemoglobin; FPG, fasting plasma glucose; BMI, body mass index; TC, total cholesterol; HDL, high density lipoprotein; LDL, low density lipoprotein; VLDL, very low density lipoprotein; TG, triglyceride; CBC, complete blood count; RBC, red blood cell; HGB, hemoglobin; HCT, hematocrit; MCV, mean corpuscular volume; MCH, Mean Corpuscular Hemoglobin; MCHC, Mean Corpuscular Hemoglobin Concentration; PSQI, pittsburgh sleep quality index; SDS, the Self-rating Depression Scale; SAS, the Self-rating Anxiety Scale; PHQ-9, the Patient Health Questionnaire-9; GAD-7, Generalized Anxiety Disorder-7; MoCA, montreal cognitive assessment; MMSE, the Mini-Mental State Examination; DST, digit span test.

### 4.2 Physiological and biochemical indices

Compared with the HC group, T2DM patients had elevated systolic blood pressure, diastolic blood pressure, VLDL, TG and RBC count, as well as decreased HDL cholesterol (all p < 0.05). No significant differences were observed in other blood lipid indicators (TC, LDL) or complete blood count indices (HGB, HCT, MCV, mean corpuscular hemoglobin [MCH], mean corpuscular hemoglobin concentration [MCHC]) (**Table 1**, **eTable S1** in **Supplementary 1**).

Regarding HbA1c, 1 patient had an HbA1c level ≤ 6.1%, while 78 patients had an HbA1c level > 6.1%. HDL distribution also differed significantly between the groups (p < 0.05). In the T2DM group, 3 patients had HDL < 0.78 mmol/L, 79 patients had HDL 0.78–1.81 mmol/L, and 1 patient had HDL > 1.81 mmol/L; in the HC group, 25 subjects had HDL 0.78–1.81 mmol/L and 3 subjects had HDL > 1.81 mmol/L.

RBC counts were assessed in 82 T2DM patients (75 normal and 7 abnormal) and in HC (36 normal).

### 4.3 Emotional symptoms

No significant between-group differences were observed in emotional symptom scores (**Table 1**; **eTable S1** in **Supplementary 1**).

### 4.4 Cognitive function

Visuospatial memory test scores, including the immediate and delayed indices, were significantly lower in patients with T2DM than in HC. No other significant between-group differences were observed in cognitive scores (**Table 1**; **eTable S1** in **Supplementary 1**).

### 4.5 Hippocampal FC

Compared with healthy controls, patients with type 2 diabetes mellitus showed significantly increased functional connectivity (FC) between the right hippocampus and the left dorsolateral prefrontal cortex (DLPFC) as well as the left superior frontal gyrus (SFG) (**Figure 1**; **eTable S2** in **Supplementary 1**).

**Figure 1:**
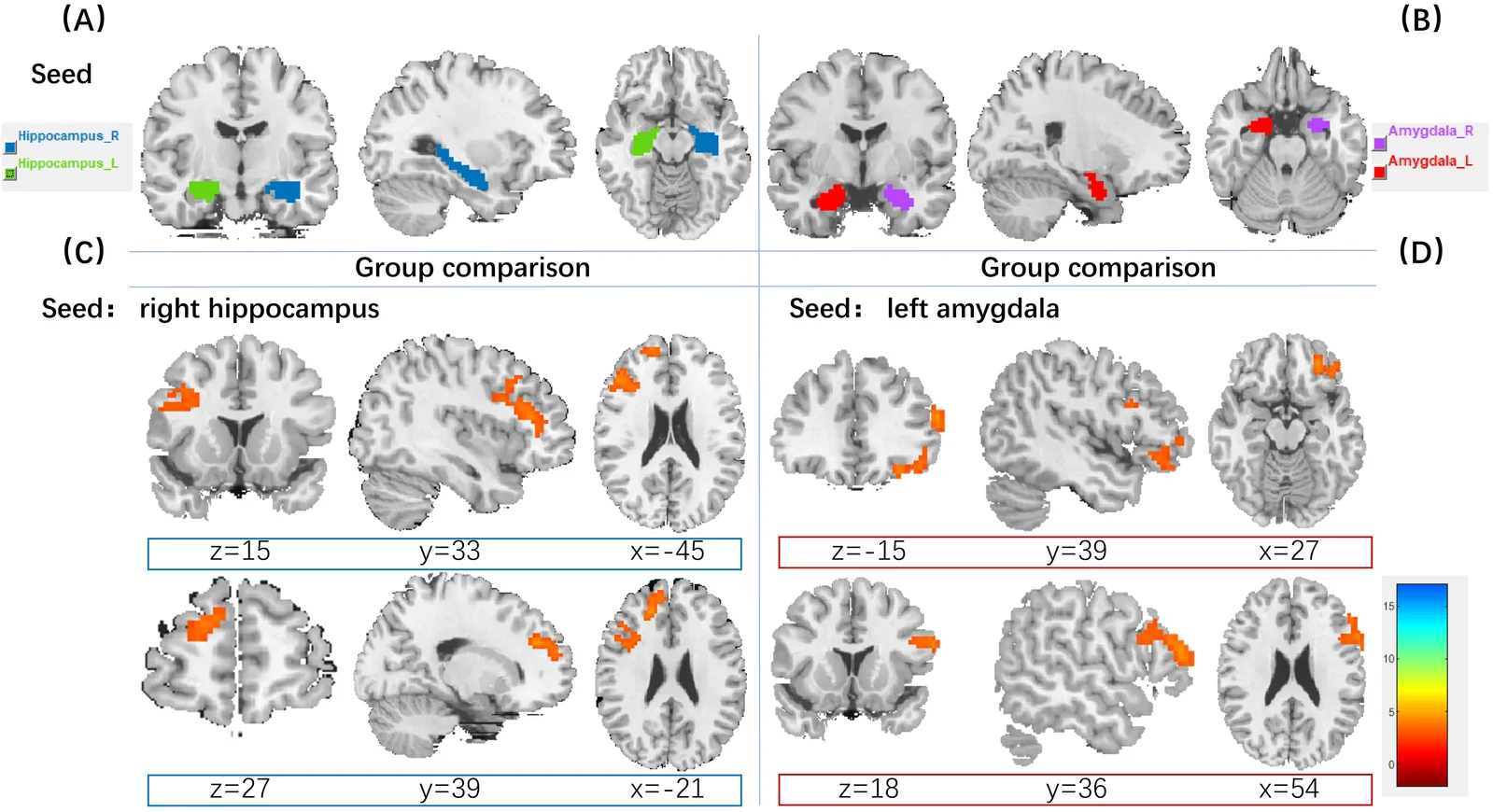
Significant differences were observed in the right hippocampus and left amygdala functional connectivity between the Tibetan plateau patients with T2DM and the Tibetan plateau HC. Thresholds were set using FWE correction at a p value of < 0.05 at the cluster level (voxel p < 0.001, cluster size > 100) and the values of X, Y, and Z represent the MNI coordinates of the axial, sagittal, and coronal positions, respectively. Color scale represents t values. **(A)** The seed of the right hippocampus. **(B)** The seed of the left amygdala. **(C)** The right hippocampus, left DLPFC, and left SFG were significantly greater in the T2DM group. **(D)** The left amygdala, right OFC, and right DLPFC were significantly greater in the T2DM group. T2DM, Type 2 diabetes mellitus; HC, healthy controls; FWE, family-wise error; MNI, montreal neurological institute; DLPFC, dorsal lateral prefrontal cortex; SFG, superior frontal gyrus; OFC, orbitofrontal cortex.

No significant difference in left hippocampal FC was observed between T2DM patients and HC.

### 4.6 Amygdala FC

Tibetan plateau T2DM patients showed significantly increased FC between the left amygdala and the right lateral orbitofrontal cortex (OFC) and right DLPFC (**Figure 1**; **eTable S2** in **Supplementary 1**).

No significant difference in right amygdala FC was observed between groups at the whole-brain level.

### 4.7 Correlation analysis

#### 4.7.1 Correlations between the right hippocampus-left DLPFC FC and clinical assessments

In the T2DM group, FC between the right hippocampus and left DLPFC correlated negatively with age (r = −0.451, p < 0.001) and positively with RBC count (r = 0.318, p = 0.004), altitude (r = 0.262, p = 0.018), visuospatial memory test-immediate score (r = 0.494, p = 0.002), visuospatial memory test-delayed score (r = 0.497, p = 0.002), DST forward (r = 0.238, p = 0.049) and DST backward (r = 0.237, p = 0.050) (**Figures 2** and **3**).

**Figure 2:**
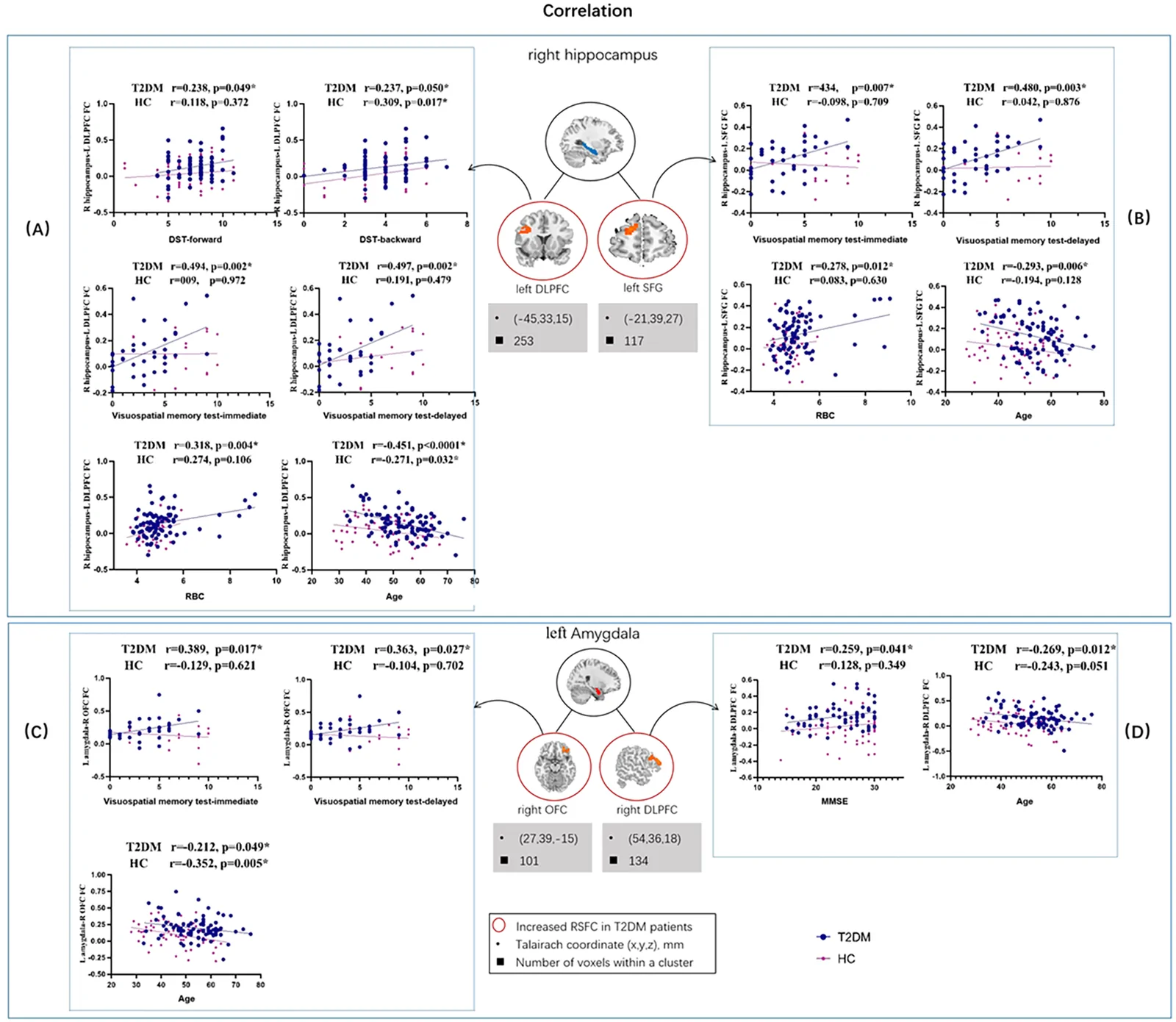
Significant correlations between the FC of the right hippocampus, the left amygdala and neuropsychological performance and different clinical variables in T2DM patients and HC. **(A)** Correlations of the FC between the right hippocampus and the left DLPFC with DST forward, DST backward, visuospatial memory test-immediate, visual memory test-delayed scores, RBC count and age. **(B)** Correlations of the FC between the right hippocampus and the left SFG with visuospatial memory test-immediate, visual memory test-delayed scores, RBC count and age. **(C)** Correlations of FC between the left amygdala and the right OFC with visuospatial memory test-immediate, visual memory test-delayed scores, and age. **(D)** Correlation of the FC between the left amygdala and the right DLPFC with MMSE score and age. FC, functional connectivity; T2DM, Type 2 diabetes mellitus; HC, healthy controls; DLPFC, dorsal lateral prefrontal cortex; SFG, superior frontal gyrus; OFC, orbitofrontal cortex; DST, digit span test; RBC, red blood cell; MoCA, montreal cognitive assessment; MMSE, the Mini-Mental State Examination.

**Figure 3:**
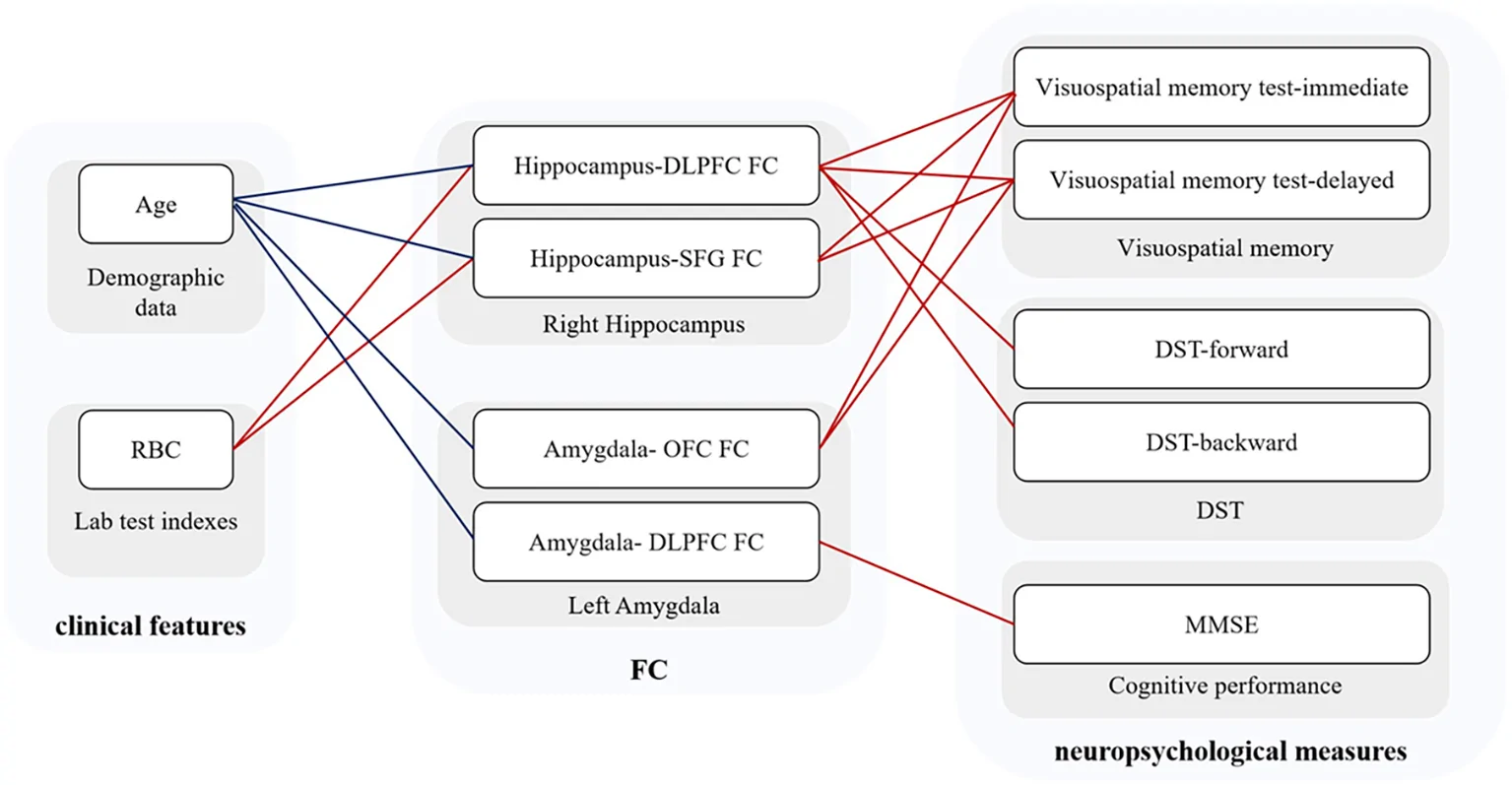
Correlations between FC and clinical features. Red: positive correlation. Blue: negative correlation.FC, functional connectivity; DLPFC, dorsal lateral prefrontal cortex; SFG, superior frontal gyrus; OFC, orbitofrontal cortex; DST, digit span test; RBC, red blood cell; MMSE, the Mini-Mental State Examination.

#### 4.7.2 Correlation between the right hippocampus-left SFG FC and clinical assessments

In the T2DM group, FC between the right hippocampus and left SFG correlated negatively with age (r = −0.293, p = 0.006) and positively with RBC count (r = 0.278, p = 0.012), but showed no correlation with blood glucose level (r = −0.104, p = 0.437).

Visuospatial memory scores, including visuospatial memory test-immediate (r = 0.434, p = 0.007) and visuospatial memory test-delayed (r = 0.480, p = 0.003), were positively correlated with FC between the right hippocampus and left SFG in the T2DM group (**Figures 2** and **3**).

#### 4.7.3 Correlations between the left amygdala-right OFC FC and clinical assessments

In the T2DM group, FC between the left amygdala and right OFC showed a nominally significant negatively correlated with age (r = −0.212, p = 0.049), but no correlation with blood glucose level (r = 0.200, p = 0.132).

Among the neuropsychological measures, this FC was positively correlated with visuospatial memory test-immediate score (r = 0.389, p = 0.017) and visuospatial memory test-delayed score (r = 0.363, p = 0.027) (**Figures 2** and **3**).

#### 4.7.4 Correlations between the left amygdala-right DLPFC FC and clinical assessments

In the T2DM group, FC between the left amygdala and right DLPFC correlated negatively with age (r = −0.269, p = 0.012), but showed no correlation with blood glucose level (r = 0.065, p = 0.629).

Among the neuropsychological measures, the FC was positively correlated with the MMSE score (r = 0.259, p = 0.041) in the T2DM group (**Figures 2** and **3**).

## 5 Discussion

This study of hippocampal and amygdala FC in Tibetan patients with T2DM revealed increased right hippocampus-DLPFC/SFG and left amygdala-OFC/DLPFC connectivity compared with HC. In exploratory bivariate analyses, hippocampal FC was associated with RBC count and cognitive performance. The observed RBC–FC associations should be interpreted cautiously because they were attenuated after adjustment for age, sex, smoking status, and residential altitude in sensitivity analyses (**eTable S4** in **Supplementary 1**).

Compared with the Tibetan plateau HC group, the T2DM group exhibited significantly greater functional connectivity (FC) between the right hippocampus-left DLPFC/SFG and the left amygdala-right OFC/DLPFC. This pattern differs from that reported in previous studies of general populations with T2DM, which found decreased FC between the right hippocampus and multiple regions, including the fusiform gyrus, middle occipital gyrus, calcarine (22), and frontal cortex (13). In the present study, we did not observe decreased hippocampal connectivity. Instead, the increased hippocampal FC with the contralateral DLPFC and SFG was associated with higher RBC count in Tibetan T2DM patients.

RBC count is positively associated with altitude in our cohort, which is consistent with prior reports (23).Conversely, insulin levels decreased with increasing altitude, along with FPG and postprandial blood glucose (6, 24), suggesting that metabolic risk factors for diabetes may differ between high-altitude and low-altitude populations. RBCs directly influence BOLD signals (14), so the RBC-FC association may partially explain this hyperconnectivity. However, its presence in patients with normal RBC count suggesting that factors other than RBC count may also be associated with the observed hyperconnectivity. The increased limbic FC observed in this high-altitude Tibetan cohort differs from the decreased FC reported in some previous low-altitude T2DM studies.

The DLPFC, a core hub of the frontoparietal network, plays a critical role in cognitive executive functions (25), and is a key brain region and therapeutic target for many disorders involving cognitive dysfunction (26, 27). Previous studies have reported executive function impairments in patients with diabetes (28). In the present study, we found that increased FC between the right hippocampus and left DLPFC in Tibetan T2DM patients was positively associated with better performance on tests of visuospatial memory and processing speed. Given that the DLPFC is critically involved in these cognitive domains, our findings suggest that T2DM-related neural changes affect memory and executive function. These findings indicate an association between enhanced hippocampal-DLPFC connectivity and cognitive performance, although the functional significance of this hyperconnectivity remains uncertain.

Beyond right hippocampus-DLPFC hyperconnectivity, T2DM patients also showed greater right hippocampus-SFG (a DMN node) FC versus HC. The SFG contributes to working memory (29) and is anatomically connected to MFG/IFG via arcuate fibers (30); we also observed hippocampus-MFG connectivity. These findings indicate disrupted frontal lobe functional integration, consistent with reports that the prefrontal cortex is vulnerable in T2DM (31, 32). Prior studies found that hippocampal FC with cognitive/visual regions correlates with reduced memory performance (33) and that SFG FC impairment relates to cognitive deficits in T2DM (31). In the present study, we found that visuospatial memory test results were positively associated with increased FC between the right hippocampus and left SFG in T2DM patients, which is consistent with the findings of previous studies. Given the cross-sectional design, the functional significance of the observed hyperconnectivity remains uncertain and requires further longitudinal investigation. Tibetan plateau patients with T2DM also showed increased FC between the left amygdala and the right OFC and right DLPFC. The amygdala is a pivotal brain region in the processing of emotions and is involved in the onset (34) and development of depression in neurobiological disease (35). Abnormalities in the amygdala have been observed in T2DM patients (36). Previous T2DM studies have shown increased FC between the left amygdala and the right cerebellum posterior lobe and between the right amygdala and the left cerebellum posterior lobe (37) and decreased amygdala FC with various regions, including the right insula, right inferior frontal gyrus, bilateral lingual gyrus, left posterior cingulate cortex (PCC) and left fusiform gyrus (37). There have been only two studies on FC in the amygdala in individuals with T2DM, and they were from the same center; our results are different from these studies, likely because of the varying research objects, probably because we studied plateau Tibetans affected by the lack of oxygen at high altitudes.

We found increased FC between the left amygdala and right OFC in T2DM patients, which was positively correlated with the visuospatial memory test score. T2DM causes a decrease in the visuospatial memory score, which affects visuospatial memory hypomnesia. As an important component of the reward system, the OFC can regulate reward and eating behavior according to incoming and outgoing information (38). Previous studies on food intake and energy balance have shown that the gray matter volume in the OFC is lower in patients with T2DM and obesity than in healthy individuals (39, 40). Functional MRI (fMRI) studies have shown that when overweight volunteers are stimulated with high-calorie food pictures, the activity in the OFC decreases significantly (41). In patients with type I diabetes mellitus, the activity of multiple brain regions, such as the OFC and amygdala, decreases during hypoglycemia (42). Previous MRI studies in animals and humans have confirmed the existence of FC between the OFC and multiple brain regions (43), including the hypothalamus and amygdala; this finding is consistent with our study. These results suggest that diabetes is indeed associated with feeding behavior.

Tibetan T2DM patients also showed increased left amygdala-right DLPFC FC, which positively correlated with MMSE scores, supporting DLPFC’s role in cognitive compensation.

In summary, Tibetan T2DM patients exhibited a consistent limbic-prefrontal hyperconnectivity pattern: increased right hippocampus-DLPFC/SFG and left amygdala-OFC/DLPFC FC. These increases positively correlated with cognitive performance (visuospatial memory, processing speed, MMSE). The cross-sectional design precludes causal interpretation. This connectivity pattern differs from that reported in some previous low-altitude T2DM studies; however, the present study cannot determine whether this difference is attributable to high-altitude exposure.

## 6 Limitations

Several limitations should be acknowledged. First, some patients had abnormal blood pressure; hypertension is a risk factor for cognitive dysfunction, but its interaction with diabetes remains unclear. Second, most prior rsFC studies in T2DM used other seeds (e.g., posterior cingulate); few examined hippocampal/amygdala FC. Our study provides new data, but replication is needed. Third, due to the cross-sectional design, we cannot determine whether the observed hyperconnectivity signals future cognitive impairment. Large-scale prospective studies are needed to establish causality. Fourth, due to the inherent constraints of *in vivo* human research, we were unable to validate our imaging findings with corresponding pathological or histological evidence from the same subjects. Future studies combining animal models or post-mortem human tissues with longitudinal imaging data will be needed to further corroborate the observed functional connectivity changes at the microscopic level. Finally, although MRI was performed within 1 week after descent, the exact interval was not recorded, which may have influenced BOLD signals.

## 7 Conclusions

We demonstrate that Tibetan T2DM patients show increased hippocampus-prefrontal and amygdala-prefrontal FC. These alterations correlate with memory and RBC count, but not glucose. Our findings link RBC count to brain functional changes in Tibetan T2DM patients and suggest a pattern of cognitive impairment distinct from Tibetan HC patients. However, given the cross-sectional design of this study, causal inferences cannot be drawn, and further longitudinal and intervention studies are needed to test this hypothesis.

## Data Availability

The raw data supporting the conclusions of this article will be made available by the authors, without undue reservation.
