# Supplementary material for: Association between red blood cell count and cortical functional connectivity in type 2 diabetes mellitus patients residing at high altitude: resting-state fMRI study in the Tibetan Plateau
Source: Front Endocrinol (Lausanne). 2026 Oct 14;17:1931880. doi: 10.3389/fendo.2026.1931880 (PMC13713285; doi:10.3389/fendo.2026.1931880)
Supplement: Supplementary file 1 [file DataSheet1.docx]

***Supplementary Material***

**1 Supplementary Data**

**eMethods 1: Emotional symptoms index**

**Pittsburgh sleep quality index (PSQI)**

The PSQI was developed in 1989 by researchers at the University of Pittsburgh. It is a self-administered questionnaire that includes 19 questions that patients answer about themselves and 5 additional optional questions that can be answered by someone who sleeps in the same room or bed with them. The questions are all intended to be answered based on patients’ sleeping patterns for the past month.(1)

**Patient health questionnaire-9 (PHQ-9)**

The Patient Health Questionnaire-9 (PHQ-9) is a self-administered, validated survey developed to assess and monitor depression severity in adults. It is derived from the longer PHQ assessment, which, in addition to depression, also includes modules that assess anxiety, somatoform, alcohol, and eating disorders. The PHQ-9 consists of nine questions that focus on the DSM-IV diagnostic criteria for major depressive disorder (MDD). Eight questions directly assess symptoms of depression, and one question assesses how any depressive symptoms have impacted a patient’s ability to function.(2)

**Generalized anxiety disorder (GAD-7)**

GAD-7 is a 7-item anxiety scale that is a useful tool with strong criterion validity for identifying probable cases of GAD. Cutoff points of 5, 10, 15 and 20 on the GAD-7 can be interpreted as representing mild, moderate, moderate/severe and severe levels of anxiety, respectively. The GAD-7 score has the best performance for identifying GAD. In addition, recent guidance in America recommended the use of the GAD-7 for assessing anxiety in cancer patients.(3, 4)

**Self-rating depression scale (SDS)**

The Zung Self-rating Depression Scale (SDS) consists of 20 items answered on a Likert-type scale. The scores for each item range from 1 to 4, and the SDS ranges from a raw score of 20 to a raw score of 80. Some items are reverse scored (i.e., they range from 4 to 1). SDS scores are classified as normal (<50), mild depression (50 to 59), moderate to marked major depression (60 to 69), and severe to extreme major depression (>70). The raw score can be converted to an SDS index score by multiplying the raw score by 1·25.(5)

**Self-rating anxiety scale (SAS)**

The Zung Self-rating Anxiety Scale (SAS) is a method of measuring levels of anxiety in patients who have anxiety-related symptoms. The scale focuses on the most common general anxiety disorders; coping with stress typically causes anxiety. The SAS is self-administered, with each response using a 4-point scale ranging from “none of the time” to “most of the time.” There are 20 questions with 15 increasing anxiety level questions and 5 decreasing anxiety questions. There are two formats, self-evaluations and clinical evaluations.(6)

**eMethods 2: Cognitive function**

**Montreal cognitive assessment (MoCA, Beijing edition)**

The Montreal Cognitive Assessment (MoCA) is a brief, 30-question test that helps healthcare professionals detect cognitive impairments very early, allowing for faster diagnosis and patient care. The MoCA is the most sensitive test available for detecting Alzheimer’s disease; it measures executive functions and multiple cognitive domains, which are important components not measured by the MMSE. The MoCA is particularly useful for detecting cognitive changes in individuals with higher education levels or for whom mild cognitive changes are the primary clinical concern.(7)

**Mini-mental state examination (MMSE)**

The Mini-Mental State Examination (MMSE) comprises a set of 11 questions that doctors and other healthcare professionals commonly use to assess cognitive impairment (problems with thinking, communication, understanding and memory). The MMSE can be used to assess 6 areas of mental abilities, including (1) orientation to time and place — knowing the date and where you are; (2) attention/concentration; (3) short-term memory (recall); (4) language skills; (5) visuospatial abilities — visual and spatial relationships between objects; and (6) ability to understand and follow instructions. The test takes approximately 5 to 10 minutes. The questions are usually the same (or very similar) regardless of who conducts the test. The maximum score for the MMSE is 30. A score of 25 or higher is considered normal. A score less than 24 is usually considered abnormal, indicating possible cognitive impairment.(8)

**Digit span test**

Digit span testing is a measure of memory span that measures the number of items, usually words or numbers, that a person can retain and recall. When numbers are used, the test is called digit repetition. It can be considered a measure of working memory (or short-term memory, depending on the psychological framework used), although other factors, such as attention and comprehension, also contribute to performance on this test. In a typical test of memory span, a list of random numbers is read out at an approximate rate of one per second. The test begins with two to three numbers, increasing until the person commits errors. Recognizable patterns (for example, 2, 4, 6, and 8) should be avoided. At the end of a sequence, the person being tested is asked to recall the items in order. The average digit span for normal adults without error is seven plus or minus two. Reverse repetition is more difficult than forward repetition and requires more processes than immediate recall.(9)

**Logical memory test**

Logical memory is a subtest from the Wechsler Memory Scale IV that comprises part of the Auditory Memory Index. A short prose passage is read to individuals, and they are asked to recall as many details as possible both immediately and after a 25-35 minutes interval. A yes/no recognition test follows the delayed. This test takes approximately 5 minutes to administer (however, sufficient time must be available to include the delay).(10)

**Visuospatial memory test**

The visuospatial memory test is a measure of visuospatial memory that distinguishes learning from recall. This test is widely used to assess the visuo-constructional ability and visual memory of individuals with neuropsychiatric disorders, and it includes copying and recall tests. By drawing complex figures, the functional decline of a patient in multiple cognitive dimensions, including attention and concentration, fine-motor coordination, visuospatial perception, nonverbal memory, planning and organization, and spatial orientation, can be assessed.

References

1.	Liu D, Kahathuduwa C, Vazsonyi AT. The Pittsburgh Sleep Quality Index (Psqi): Psychometric and Clinical Risk Score Applications among College Students. *Psychological assessment* (2021) 33(9):816-26. Epub 2021/05/21. doi: 10.1037/pas0001027.

2.	Blackwell TL, McDermott AN. Review of Patient Health Questionnaire–9 (Phq-9). *Rehabil Couns Bull* (2014) 57(4):246-8. doi: 10.1177/0034355213515305.

3.	Spitzer RL, Kroenke K, Williams JB, Löwe B. A Brief Measure for Assessing Generalized Anxiety Disorder: The Gad-7. *Archives of internal medicine* (2006) 166(10):1092-7. Epub 2006/05/24. doi: 10.1001/archinte.166.10.1092.

4.	Plummer F, Manea L, Trepel D, McMillan D. Screening for Anxiety Disorders with the Gad-7 and Gad-2: A Systematic Review and Diagnostic Metaanalysis. *General hospital psychiatry* (2016) 39:24-31. Epub 2016/01/01. doi: 10.1016/j.genhosppsych.2015.11.005.

5.	Andersen BL, DeRubeis RJ, Berman BS, Gruman J, Champion VL, Massie MJ, et al. Screening, Assessment, and Care of Anxiety and Depressive Symptoms in Adults with Cancer: An American Society of Clinical Oncology Guideline Adaptation. *Journal of clinical oncology : official journal of the American Society of Clinical Oncology* (2014) 32(15):1605-19. Epub 2014/04/16. doi: 10.1200/jco.2013.52.4611.

6.	Dunstan DA, Scott N. Norms for Zung's Self-Rating Anxiety Scale. *BMC psychiatry* (2020) 20(1):90. Epub 2020/03/01. doi: 10.1186/s12888-019-2427-6.

7.	Nasreddine ZS, Phillips NA, Bédirian V, Charbonneau S, Whitehead V, Collin I, et al. The Montreal Cognitive Assessment, Moca: A Brief Screening Tool for Mild Cognitive Impairment. *Journal of the American Geriatrics Society* (2005) 53(4):695-9. Epub 2005/04/09. doi: 10.1111/j.1532-5415.2005.53221.x.

8.	Folstein MF, Folstein SE, McHugh PR. "Mini-Mental State". A Practical Method for Grading the Cognitive State of Patients for the Clinician. *J Psychiatr Res* (1975) 12(3):189-98. Epub 1975/11/01. doi: 10.1016/0022-3956(75)90026-6.

9.	Leung JL, Lee GT, Lam YH, Chan RC, Wu JY. The Use of the Digit Span Test in Screening for Cognitive Impairment in Acute Medical Inpatients. *International psychogeriatrics* (2011) 23(10):1569-74. Epub 2011/07/07. doi: 10.1017/s1041610211000792.

10.	Wechsler D. Wechsler Memory Scale-Fourth Edition (Wms-Iv) Technical and Interpretive Manual. (2008).

**2 Supplementary Figures and Tables**

**Table S1 Detailed breakdown of Table 1：Layered analysis of clinical data, psychological symptoms and neuropsychiatric test results in T2DM patients and HC**

|  | **T2DM group**  **(n = 87)** | **HC group**  **(n = 63)** | **p value** |
| --- | --- | --- | --- |
| HbA1c (%) |  |  | NA |
| (≤ 6·1) | 1 | NA |  |
| (> 6·1) | 78 | NA |  |
| FPG (mmol/L) |  |  | NA |
| (≤ 6·11) | 15 | NA |  |
| (>6·11) | 43 | NA |  |
| Systolic blood pressure (mmHg) |  |  | 0·002* |
| (≤ 140 mmHg) | 64 | 44 |  |
| (> 140 mmHg) | 20 | 1 |  |
| Diastolic blood pressure (mmHg) |  |  | 0·002* |
| (≤ 90 mmHg) | 65 | 44 |  |
| (> 90 mmHg) | 19 | 1 |  |
| Cholesterol |  |  |  |
| TC (mmol/L) |  |  | 0·31 |
| (< 3·6) | 19 | 3 |  |
| (3·6-6·5) | 63 | 25 |  |
| (> 6·5) | 1 | NA |  |
| HDL (mmol/L) |  |  | 0·042* |
| (< 0·78) | 3 | NA |  |
| (0·78-1·81) | 79 | 25 |  |
| (> 1·81) | 1 | 3 |  |
| LDL (mmol/L) |  |  | 0·33 |
| (≤ 4) | 81 | 31 |  |
| (> 4) | 2 | 2 |  |
| VLDL (mmol/L) |  |  | 0·47 |
| (< 0·26) | 9 | 2 |  |
| (0·26-1·90) | 72 | 31 |  |
| (> 1·90) | 2 | NA |  |
| TG (mmol/L) |  |  | 0·083 |
| (≤ 1·92) | 69 | 26 |  |
| (> 1·92) | 14 | 1 |  |
| RBC (10^12^/L) |  |  | 0·071 |
| (< 6·5) | 75 | 36 |  |
| (≥ 6·5) | 7 | NA |  |
| HGB (g/L) |  |  | 0·124 |
| (< 130) | 10 | 7 |  |
| (130-175) | 60 | 28 |  |
| (> 175) | 12 | 1 |  |
| HCT (%) |  |  | 0·055 |
| (< 40) | 11 | 8 |  |
| (40-50) | 56 | 27 |  |
| (> 50) | 15 | 1 |  |
| MCV (fL) |  |  | 0·052 |
| (< 82) | 6 | 2 |  |
| (82-100) | 74 | 29 |  |
| (> 100) | 2 | 5 |  |
| MCH (pg) |  |  | 0·32 |
| (< 27) | 10 | 5 |  |
| (27-34) | 70 | 28 |  |
| (> 34) | 2 | 3 |  |
| MCHC (g/L) |  |  | 0·79 |
| (< 316) | 10 | 4 |  |
| (316-354) | 71 | 32 |  |
| (> 354) | 1 | NA |  |
| PSQI |  |  | 0·12 |
| Very good (0-5) | 46 | 27 |  |
| Fairly good (6-10) | 20 | 18 |  |
| Fairly bad (11-15) | 5 | 11 |  |
| Very bad (16-21) | 2 | 1 |  |
| SDS |  |  | 0·62 |
| Normal (< 0·5) | 60 | 46 |  |
| Slight to mild depression (0·5-0·59) | 7 | 7 |  |
| Moderate to severe depression (0·6-0·69) | 3 | 2 |  |
| Severe depression (≥ 0·7) | 1 | 3 |  |
| SAS |  |  | 0·31 |
| Normal (< 50) | 67 | 53 |  |
| Mild anxiety (50-59) | 2 | 4 |  |
| Moderate anxiety (60-69) | 0 | 1 |  |
| Severe anxiety (≥ 70) | 0 | 0 |  |
| PHQ-9 |  |  | 0·85 |
| Normal (0-4) | 53 | 43 |  |
| Mild depression (5-9) | 10 | 11 |  |
| Moderate depression (10-14) | 4 | 2 |  |
| Moderate to severe depression (15-19) | 2 | 2 |  |
| Severe depression (20-27) | 0 | 0 |  |
| GAD-7 |  |  | 0·52 |
| Normal (0-4) | 53 | 44 |  |
| Slight anxiety (5-9) | 13 | 12 |  |
| Moderate anxiety (10-13) | 2 | 0 |  |
| Moderate to severe anxiety (14-18) | 1 | 2 |  |
| Severe anxiety (19-21) | 0 | 0 |  |
| MMSE |  |  | 0·15 |
| Mild (≥ 21) | 46 | 47 |  |
| Moderate (10-20) | 17 | 9 |  |
| Severe (≤ 9) | 0 | 0 |  |
| MoCA |  |  | 0·49 |
| Normal (≥ 26) | 12 | 13 |  |
| MCI (18-26) | 31 | 31 |  |
| Moderate (10-17) | 15 | 11 |  |
| Severe (< 10) | 2 | 0 |  |

NA, not applicable. *p value < 0·05. T2DM: Type 2 diabetes mellitus; HC: healthy controls; HbA1c, glycosylated hemoglobin; FPG, fasting plasma glucose; TC, total cholesterol; HDL, high density lipoprotein; LDL, low density lipoprotein; VLDL, very low density lipoprotein; TG, triglyceride; CBC, complete blood count; RBC, red blood cell; HGB, hemoglobin; HCT, hematocrit; MCV, mean corpuscular volume; MCH,mean corpuscular hemoglobin; MCHC, mean corpuscular hemoglobin concentration; PSQI, pittsburgh sleep quality index; SDS, the Self-rating Depression Scale; SAS, the Self-rating Anxiety Scale; PHQ-9, the Patient Health Questionnaire-9; GAD-7, Generalized Anxiety Disorder-7; MoCA, montreal cognitive assessment; MMSE, the Mini-Mental State Examination.

**Table S2 Aberrant functional connectivity in the T2DM patient group compared with the HC group**

| **Seed** | **Brain regions** | **Peak MNI coordinates** | | | **Cluster size** | **T** | **P (FWE)** |
| --- | --- | --- | --- | --- | --- | --- | --- |
|  |  | **X** | **Y** | **Z** |  |  |  |
| Right hippocampus | Left dorsal lateral prefrontal cortex | -45 | 33 | 15 | 253 | 5.35 | < 0.001* |
|  | Left superior frontal gyrus | -21 | 39 | 27 | 117 | 4.73 | 0.006* |
| Left amygdala | Right orbitofrontal cortex | 27 | 39 | -15 | 101 | 4.62 | 0.011* |
|  | Right dorsal lateral prefrontal cortex | 54 | 36 | 18 | 134 | 4.44 | 0.003* |

T2DM: Type 2 diabetes mellitus; HC: healthy controls; MNI: Montreal Neurological Institute; FWE: family-wise error. *: p< 0.05.

**Table S3 Correlations between altered hippocampal/amygdala functional connectivity and clinical variables in patients with T2DM**

| Variable | Right hippocampus  -left DLPFC | Right hippocampus  -left SFG | Left amygdala  -right OFC | Left amygdala  -right DLPFC |
| --- | --- | --- | --- | --- |
| Demographic/exposure |  |  |  |  |
| Age | N = 87 , r = -0.451,  P<0.0001,q= 0.002* | N =87 , r = -0.293,  p=0.006,q =0.048* | N =87, r= -0.212,  p =0.049, q = 0.173 | N = 87, r = -0.269,  p= 0.012,q = 0.064 |
| Altitude | N = 86, r = 0.031,  p = 0.778,q = 0.847 | N = 86, r = 0.038,  p =0.727,q = 0.847 | N = 86, r = -0.085,  p = 0.44, q = 0.704 | N = 86, r = -0.028,  p = 0.794,q = 0.847 |
| Illness duration | N = 87, r = -0.199,  p = 0.065,q = 0.173 | N = 87, r = -0.073,  p =0.499,q = 0.726 | N = 87, r = -0.043,  p = 0.695,q = 0.847 | N = 87, r= -0.0188,  p =0.863,q = 0.863 |
| BMI | N = 87, r = 0.141 ,  p = 0.201, q = 0.459 | N = 87, r = 0.206,  p =0.060,q = 0.173 | N = 87, r = -0.113,  p =0.301, q = 0.602 | N = 87, r = -0.105,  p=0.342, q = 0.608 |
| Hematological |  |  |  |  |
| RBC | N = 82, r = 0.318,  p =0.004, q = 0.036* | N = 82, r = 0.278,  P=0.012,q= 0.036* | N = 82, r = 0.122,  p =0.277, q = 0.369 | N = 82, r = 0.185,  p =0.096,q = 0.165 |
| HGB | N = 82, r = 0.283,  p = 0.010,q = 0.036* | N = 82, r = 0.247,  p =0.025,q = 0.050 | N = 82, r = 0.084,  p = 0.456, q =0.497 | N = 82, r = 0.143,  p = 0.199,q =0.299 |
| HCT | N = 82, r = 0.279,  p = 0.011,q = 0.036* | N = 82, r = 0.257,  p=0.020,q= 0.048* | N = 82, r = 0.066,  p =0.559, q = 0.559 | N = 82, r = 0.112,  p =0.315,q = 0.378 |
| Metabolic/vascular |  |  |  |  |
| HbA1c | N = 83 , r = -0.010,  p = 0.931,q =0.958 | N = 83, r = 0.116,  p =0.310,q = 0.836 | N = 83, r = 0.093,  p = 0.413, q =0.836 | N = 83, r = 0.142,  p = 0.211,q =0.836 |
| FPG | N = 83, r = 0.026,  p =0.845,q = 0.932 | N = 83, r = -0.104,  p =0.437,q = 0.836 | N = 83, r = 0.200,  p = 0.132, q =0.836 | N = 83, r = 0.065,  p = 0.629,q =0.840 |
| TC | N = 83, r = 0.151,  p = 0.174,q =0.836 | N = 83, r = 0.112,  p = 0.315,q =0.836 | N = 83, r = -0.073,  p = 0.511, q =0.836 | N = 83, r = -0.052,  p = 0.640,q =0.840 |
| HDL | N = 83, r = 0.075,  p = 0.496,q =0.836 | N = 83, r = 0.077,  p = 0.487,q =0.836 | N = 83, r = -0.109,  p = 0.326, q =0.840 | N = 83 , r = -0.136,  p = 0.222,q =0.836 |
| LDL | N = 83, r = 0.195,  p = 0.078,q =0.836 | N = 83, r = 0.068,  p = 0.539,q =0.836 | N = 83, r = -0.002,  p = 0.984, q =0.836 | N = 83, r = -0.021,  p= 0.854, q =0.836 |
| VLDL | N = 83, r = -0.070,  p = 0.532,q =0.836 | N = 83, r = 0.096,  p = 0.387,q =0.984 | N = 83, r = -0.118,  p = 0.288, q =0.931 | N = 83, r = -0.097,  p = 0.385, q =0.836 |
| TG | N = 83, r = -0.065,  p = 0.557, q =0.836 | N = 83, r = 0.099,  p = 0.369,q =0.836 | N = 83, r = -0.050,  p = 0.653, q = 0.840 | N = 83, r = 0.054,  p = 0.629, q = 0.840 |
| SBP | N = 84, r = -0.078,  p = 0.482,q = 0.836 | N = 84, r = 0.046,  p =0.681,q = 0.845 | N = 84, r = -0.118,  p = 0.284, q =0.836 | N = 84, r = -0.090,  p = 0.415,q =0.836 |
| DBP | N = 84, r = 0.031,  p = 0.778,q =0.931 | N = 84, r = 0.013,  p = 0.907,q =0.958 | N = 84, r = -0.089,  p =0.421, q = 0.836 | N = 84, r = -0.021,  p = 0.852,q =0.932 |
| Cognitive |  |  |  |  |
| Visuospatial memory-immediate | N = 69, r = 0.494,  p =0.002, q = 0.032* | N = 69, r = 0.43,  p =0.007, q = 0.056 | N = 69, r = 0.389,  p =0.017, q = 0.109 | N = 69, r = 0.224,  p =0.182,q = 0.364 |
| Visuospatial  memory-delayed | N = 69, r = 0.497,  p = 0.002, q =0.032* | N = 69, r = 0.480,  p=0.003,q =0.032* | N = 69, r = 0.363,  p = 0.027, q =0.144 | N = 69, r = 0.261,  p =0.119, q =0.290 |
| Logical memory  test-immediate | N = 73, r = 0.118,  p = 0.414,q =0.587 | N = 73, r = 0.178,  p = 0.215,q =0.405 | N = 73, r = 0.053,  p = 0.714, q =0.816 | N = 73, r = -0.035,  p =0.810, q =0.864 |
| Logical memory test-delayed | N = 73, r = 0.142,  p = 0.325,q =0.495 | N = 73, r = 0.208,  p = 0.127,q =0.290 | N = 73, r = 0.076,  p = 0.598, q =0.765 | N = 73, r = -0.013,  p = 0.926,q =0.926 |
| DST forward | N = 73, r = 0.238,  p = 0.049,q =0.178 | N = 73, r = 0.209,  p = 0.084,q =0.269 | N =73, r = 0.035,  p = 0.778, q =0.858 | N = 73, r = 0.123,  p = 0.315,q =0.495 |
| DST backward | N = 73, r = 0.237,  p = 0.05,q =0.178 | N = 73, r = 0.191,  p = 0.116,q =0.290 | N = 73, r = 0.098,  p = 0.422, q =0.587 | N = 73, r = -0.011,  p= 0.926, q =0.926 |
| MMSE | N = 63, r = 0.142,  p = 0.268,q =0.476 | N = 63, r = 0.056,  p = 0.663,q =0.816 | N = 63, r = 0.048,  p = 0.710, q =0.816 | N = 63, r = 0.259,  p = 0.041,q =0.178 |
| MoCA | N = 60, r = 0.185,  p = 0.157,q =0.335 | N = 60, r = 0.131,  p = 0.319,q =0.495 | N = 60, r = 0.072,  p = 0.583, q =0.765 | N =60, r = 0.212,  p = 0.105,q =0.290 |
| Emotional/Sleep measures |  |  |  |  |
| PSQI | N = 73, r = 0.002,  p = 0.990,q =0.990 | N = 73, r = 0.143,  p = 0.227,q =0.982 | N = 73, r = -0.042,  p = 0.722, q =0.982 | N = 73, r = 0.034,  p = 0.773,q =0.982 |
| PHQ-9 | N = 69, r = -0.080,  p = 0.514,q =0.982 | N = 69, r = 0.040,  p = 0.742,q =0.982 | N = 69, r = -0.013,  p = 0.912, q =0.982 | N = 69, r = -0.037,  p =0.763, q =0.982 |
| GAD-7 | N = 69, r = -0.024,  p = 0.841,q =0.982 | N = 69, r = 0.151,  p = 0.216,q =0.982 | N = 69, r = 0.042,  p = 0.730, q =0.982 | N = 69, r = 0.014,  p =0.907, q =0.982 |
| SDS | N = 71, r = -0.146,  p = 0.224,q =0.982 | N = 71, r = 0.047,  p = 0.695,q =0.982 | N = 71, r = -0.061,  p = 0.611, q =0.982 | N = 71, r = -0.073,  p = 0.541,q =0.982 |
| SAS | N = 69, r =-0.090,  p = 0.458,q =0.982 | N = 69, r = -0.089,  p = 0.467,q =0.982 | N = 69, r = -0.039,  p = 0.749, q =0.982 | N = 69, r = 0.096,  p = 0.435,q =0.982 |
| PSQI | N = 73, r = 0.002,  p = 0.990,q =0.990 | N = 73, r = 0.143,  p = 0.227,q =0.982 | N = 73, r = -0.042,  p = 0.722, q =0.982 | N = 73, r = 0.034,  p = 0.773,q =0.982 |
| PHQ-9 | N = 69, r = -0.080,  p = 0.514,q =0.982 | N = 69, r = 0.040,  p = 0.742,q =0.982 | N = 69, r = -0.013,  p = 0.912, q =0.982 | N = 69, r = -0.037,  p =0.763, q =0.982 |

Pearson’s correlation analyses were performed to examine associations between hippocampal/amygdala functional connectivity and clinical variables. N denotes the valid sample size for each correlation test. r, Pearson correlation coefficient; p, uncorrected P value; q, P value adjusted for multiple comparisons using the Benjamini–Hochberg false discovery rate (FDR) method. Asterisk (*) indicates statistically significant correlation after FDR correction (q < 0.05). DLPFC, dorsolateral prefrontal cortex; SFG, superior frontal gyrus; OFC, orbitofrontal cortex; RBC, red blood cell; HGB, hemoglobin; HCT, hematocrit; HDL, high-density lipoprotein; LDL, low-density lipoprotein; VLDL, very low-density lipoprotein; FPG, fasting plasma glucose; SBP, systolic blood pressure; DBP, diastolic blood pressure; DST, Digit Span Test; MMSE, Mini-Mental State Examination; MoCA, Montreal Cognitive Assessment; PSQI, Pittsburgh Sleep Quality Index; PHQ-9, Patient Health Questionnaire-9; GAD-7, Generalized Anxiety Disorder-7; SDS, Self-Rating Depression Scale; SAS, Self-Rating Anxiety Scale.

**Table S4 Sensitivity analyses of associations between RBC count and hippocampal functional connectivity in patients with T2DM.**

| Functional connectivity | n | Partial r | P value | FDR-adjusted q |
| --- | --- | --- | --- | --- |
| Right hippocampus-left DLPFC | 80 | 0.153 | 0.186 | 0.186 |
| Right hippocampus-left SFG | 80 | 0.184 | 0.112 | 0.186 |

Note: Partial correlation analyses were performed in patients with T2DM and adjusted for age, sex, smoking status, and residential altitude. P values are two-sided. The Benjamini–Hochberg false discovery rate (FDR) procedure was applied across the two sensitivity analyses. DLPFC, dorsolateral prefrontal cortex; SFG, superior frontal gyrus; RBC, red blood cell; T2DM, type 2 diabetes mellitus.
